# P-1436. Respiratory syncytial virus vaccine uptake among Medicare fee-for-service beneficiaries, 2023-2025

**DOI:** 10.1093/ofid/ofaf695.1623

**Published:** 2026-01-11

**Authors:** Amanda B Payne, Josephine Mak, Shannon Novosad, Heng-Ming Sung, Yue Zhang, Ryan E Wiegand, Andrea J Chavez, Danica J Gomes, Morgan Najdowski, Yixin Jiao, Yenlin Lai, Yangping Chen, Yoganand Chillarige, Kelly M Hatfield, Sujan Reddy, Amber Kautz, Michael Melgar, Ruth Link-Gelles, Amadea Britton

**Affiliations:** CDC, Atlanta, Georgia; Division of Healthcare Quality Promotion, Centers for Disease Control and Prevention, Atlanta, Georgia; Centers for Disease Control and Prevention; Acumen LLC, Burlingame, California; Acumen LLC, Burlingame, California; Centers for Disease Control and Prevention; Acumen, LLC, Arlington, Virginia; Centers for Disease Control and Prevention; CDC, Atlanta, Georgia; Acumen LLC, Burlingame, California; Acumen, LLC, Arlington, Virginia; Acumen, LLC, Arlington, Virginia; Acumen LLC, Burlingame, California; Centers for Disease Control and Prevention; CDC, Atlanta, Georgia; Centers for Disease Control and Prevention; Centers for Disease Control and Prevention; Centers for Disease Control and Prevention; Centers for Disease Control and Prevention , Atlanta, GA

## Abstract

**Background:**

Respiratory syncytial virus (RSV) causes substantial morbidity and mortality in older adults. In June 2023, CDC’s Advisory Committee on Immunization Practices recommended adults aged ≥60 years receive one RSV vaccine dose under shared clinical decision making. The recommendation was updated in June 2024 as follows: adults aged ≥75 years and adults aged 60–74 years at increased risk for severe RSV disease are recommended to receive one RSV vaccine dose. Monitoring RSV vaccine uptake informs vaccine effectiveness studies and the impact of updated vaccine recommendations.

**Figure 1:**
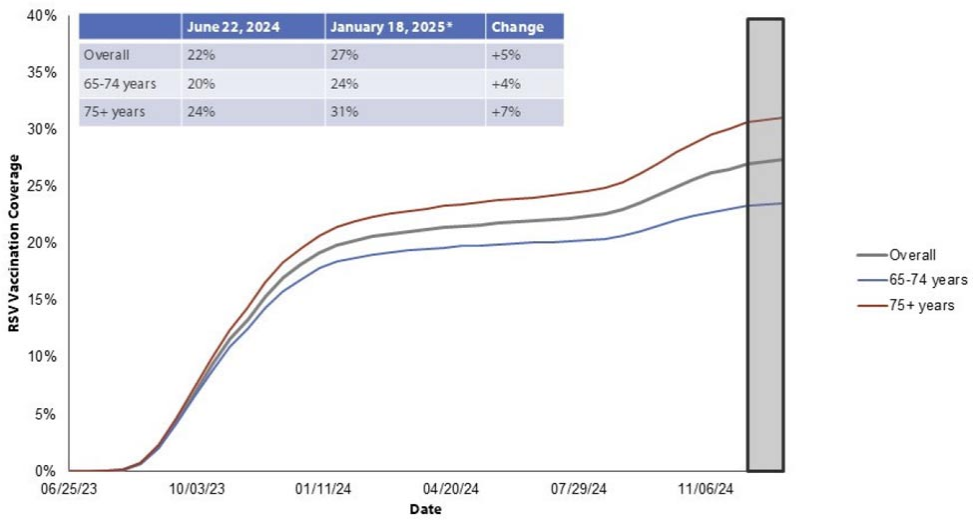
Biweekly cumulative RSV vaccination coverage, by age group, community-dwelling Medicare Fee-For-Service beneficiaries aged ≥65 Years and enrolled in a Part D plan, United States Fee-for-Service: enrolled in Medicare Parts A/B (and not Part C) for 365 days prior to reporting period. *6-week reporting lag; data may be incomplete after December 7, 2024.

**Figure 2:**
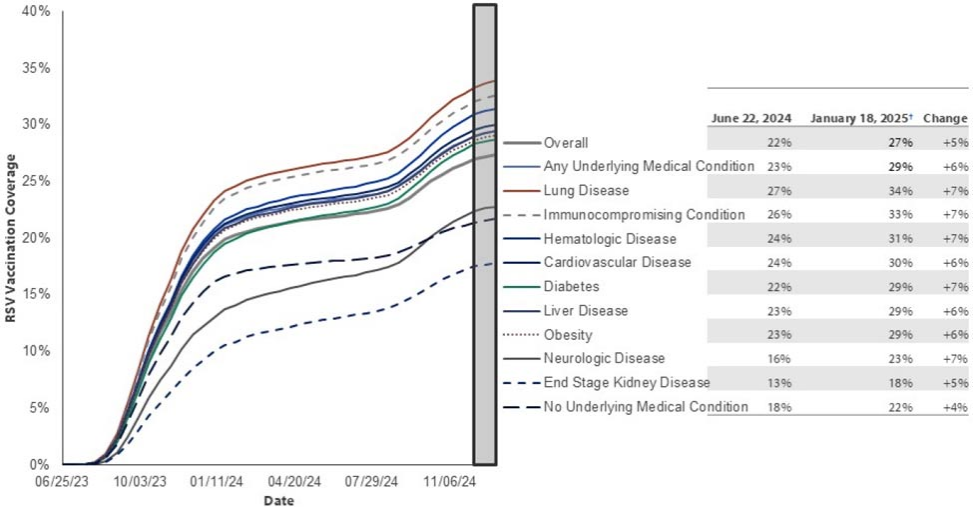
Biweekly cumulative RSV vaccination coverage, by underlying medical condition*, Community-dwelling Medicare Fee-for-Service beneficiaries aged ≥65 years and enrolled in Part D plan, United States Fee-for-Service: enrolled in Medicare Parts A/B for 365 days prior to reporting period. * Conditions included lung disease, hematologic disease, cardiovascular disease, diabetes associated with organ damage, liver disease, obesity, neurologic disease, end stage kidney disease, and immunocompromising conditions. Presence of underlying medical condition (other than immunocompromising conditions) was defined as ≥1 claim in prior 365 days listing ICD-10 diagnosis code corresponding to condition. Presence of immunocompromising condition was defined as ≥2 encounters in prior 183 days with a diagnosis code. Immunocompromising conditions included hematologic malignancy, solid malignancy, transplant, rheumatologic/inflammatory conditions, other intrinsic immune conditions, or HIV. End stage kidney disease was defined as ≥1 claim for dialysis encounter in prior 90 days (excluding acute kidney injury). | †6-week reporting lag; data may be incomplete after December 7, 2024.

**Methods:**

RSV vaccine uptake was assessed in biweekly periods during June 2023 through January 2025. All Medicare beneficiaries aged ≥65 years, enrolled in Medicare Part D since June 21, 2023, through the last Saturday of a biweekly period, and continuously enrolled in Parts A and B in the 365 days prior to the first day of a biweekly period were included. Vaccination status was determined through Medicare Part D. Beneficiaries who received an RSV vaccine prior to June 21, 2023, were excluded. Vaccine uptake was assessed by age group, nursing home residence, and underlying medical condition (UMC) status.

**Figure 3:**
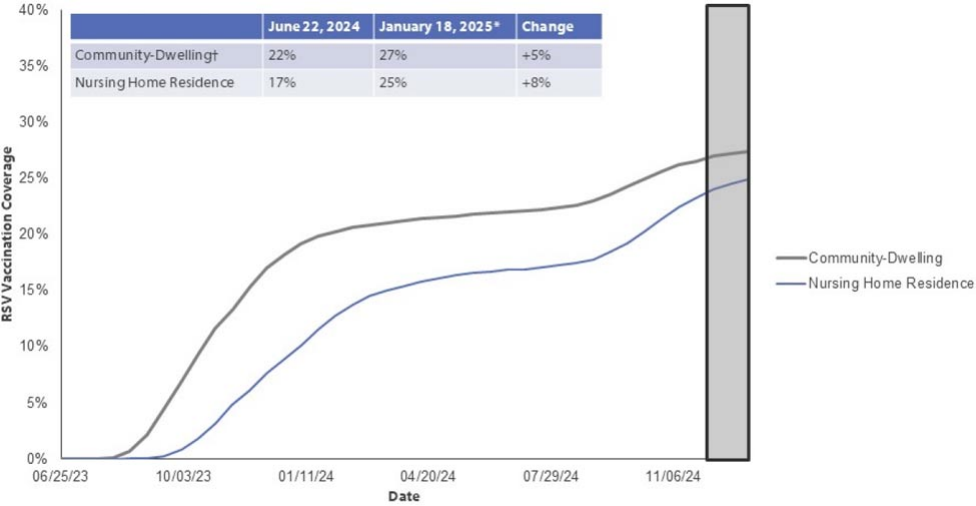
Biweekly cumulative RSV vaccination coverage, by community-dwelling or nursing home-dwelling Medicare Fee-for-Service beneficiaries aged ≥65 years and enrolled in Part D plan, United States Fee-for-Service: enrolled in Medicare Parts A/B for 365 days prior to reporting period. *6-week reporting lag; data may be incomplete after December 7, 2024. | †Regardless of age or underlying medical condition status.

**Results:**

An average of 16,176,382 Medicare Fee-For-Service beneficiaries were included in each biweekly period. RSV vaccine uptake sharply increased during June 2023 to January 2024 (19%); uptake was slower during June 2024 to January 2025 (5%) (Figure 1). By January 5–18, 2025, 27% of eligible beneficiaries had received the RSV vaccine. Uptake was higher among those aged ≥75 years (31%) compared to those aged 65-74 years old (24%). RSV vaccine uptake varied by UMC category, with beneficiaries with lung disease having the highest uptake (34%) and beneficiaries with end stage kidney disease having the lowest uptake (18%) (Figure 2). RSV vaccination coverage was consistently lower among nursing home residents compared with community-dwelling beneficiaries (Figure 3).

**Conclusion:**

RSV vaccine uptake among Medicare Fee-For-Service beneficiaries aged ≥65 years was modest after updated RSV vaccine recommendations. Differences in RSV vaccine uptake were observed by age group, nursing home residence, and UMCs.

**Disclosures:**

Ryan E. Wiegand, PhD, Merck & Co., Inc.: Stocks/Bonds (Public Company)|Sanofi S.A.: Stocks/Bonds (Public Company)

